# Cognitive Returns to Having Better Educated Teachers: Evidence from the China Education Panel Survey

**DOI:** 10.3390/jintelligence9040060

**Published:** 2021-12-03

**Authors:** Ji Liu

**Affiliations:** Faculty of Education, Shaanxi Normal University, Xi’an 710062, China; jiliu@snnu.edu.cn

**Keywords:** teacher education, student learning, cognitive performance, fixed-effects, China

## Abstract

Teachers’ own level of human capital development is commonly believed to be deterministic for the quality and effectiveness of their instruction and management in the classroom. Yet, there still exists an international debate on whether better educated teachers contribute to students’ cognitive development. Leveraging a random class-assignment subsample (N = 3436) from a nationally representative teacher-student linked dataset in China, this study reassesses the ongoing contention regarding the value of teacher education. By linking differences in teachers’ own educational attainment levels across different subjects of instruction to variation in seventh grade students’ Chinese, Math and English test scores using student fixed-effect models, this study quantifies the cognitive returns attributable to better educated teachers, in student learning terms. Findings show that teachers with at least a bachelor’s degree contribute substantially to student learning compared to those who are less qualified, by as much as 0.069 SDs or about two additional months of learning over a typical academic year. Additional sensitivity analyses suggest that this observed effect is robust to model specifications, and is consistent for students from different backgrounds.

## 1. Introduction

Across the globe, there seems to be converging consensus that the quality of teachers holds central weight to making substantive progress in educational development. Notably, the quality of teachers is cited as the single most critical external factor determining student cognitive development (see Glazerman et al. 2010), with long-term implications on adulthood success and intergenerational outcomes (Chetty et al. 2014). Rigorous research in the United States has indicated that students’ cognitive function can develop three times as much with a high-quality teacher as opposed to studying with a less effective teacher (Rockoff 2004). More important, such links between the quality of teachers and student learning outcomes have been shown to be more influential in low- and middle-income countries, where school factors play decisive roles in supporting student’s cognitive growth (Bau and Das 2017; Liu and Steiner-Khamsi 2020).

Coinciding with rising policy attention in attracting talented individuals to join the teacher workforce, teacher education researchers and teacher policy makers have become increasingly interested in understanding how observable traits are related to student learning improvements (Liu and Xie 2021). In theoretical terms, holding a higher level of educational attainment can imply either a better state of a teacher’s own level of human capital development, or can act as a positive signal of adept innate ability, academic motivation, and cognitive skills development compared to those teachers without such credentials. To that end, a teacher’s better state of their own human capital development may reflect more effective instructional craft, higher efficiency in classroom management, and greater creativity to bolster learning (Liu 2021a).

However, a large body of prior research shows that a teacher’s own educational background poorly proxies their contribution towards student learning (see Hanushek and Rivkin 2006; Harris and Sass 2007; Winters et al. 2012). On the one hand, scholars contend that a teacher’s educational background captures a wide range of accumulated human capital types, including academic proficiency, pedagogical knowledge, content and curriculum training (Kennedy et al. 2008), all of which are reasonably expected to positively influence teacher preparedness and instructional effectiveness, which act as key mediators influencing student learning. On the other hand, teacher education is under siege, since many studies argue that degrees and diplomas are noisy signals of teacher preparedness, and it is uninformative at best in predicting instructional effectiveness in the classroom. More recently, new evidence has put in question policy efforts that are designed to revamp teacher education systems and improve teacher education programs (Bastian 2019). 

Against the backdrop of this ongoing international contention, one education system that could benefit from a rigorous evaluation of the value of teacher education is China, which has rapidly professionalized its teaching force. In recent decades, a national policy impetus has focused on modernizing the education system (Liu 2019), which included a movement to bachelorize teachers by mandating longer and higher-quality teacher preparation at the bachelor’s level. To this end, holding a bachelor’s degree has played an increasingly important role in both licensure and job search in the Chinese teacher labor market (Zhu and Han 2006), and has led to the rapid rise of bachelor degree holders in the teaching force (Liu 2021c). While teacher education reforms have been rapidly implemented, the policy movement to rapidly bachelorize China’s teaching force has not been without its critics. For instance, Hu (2015) argues that there is no convincing evidence that those teachers without bachelor’s degrees are instructionally ineffective, nor there is convincing research that indicates obtaining a higher degree level can significantly improve student learning. Instead of developing actual pedagogical skills, Hu (2015) hypothesizes that the ‘bachelorization movement’ brings no obvious benefit to instruction, and is a natural consequence of diploma inflation. Coincidentally, some studies reveal that while more teacher candidates enroll in bachelor preparation programs, the norms of instruction remained virtually unchanged in many teacher preparation institutions, putting such improvements in further doubt (Zhou et al. 2011). 

Informed by literature on the importance of teacher education and instructional effectiveness, this present study sought to examine to what extent teacher educational attainment matters for student learning outcomes in China. Specifically, there were two inter-related research questions under exploration: (1) Are teachers with bachelor’s degrees (B.A.) more effective than teachers with less educational attainment? (2) Is this impact on student learning robust and heterogeneous? To answer these questions, this study leveraged the 2013-15 China Education Panel Survey (CEPS) seventh grade cohort dataset, and conceptualized the impact of teacher educational attainment as their marginal contribution towards student learning. In operational terms, this study exploited variation in teachers’ educational attainment across different subjects of instruction and related such differences to variation in student subject-test scores, while holding other relevant factors constant. 

## 2. Data and Methodology

The identification of causal relationships between observable teacher characteristics and student learning outcomes often suffers from selection bias issues, where there may be non-random sorting between students and schools, as well as self-selection bias within schools between students and teachers. To address such concerns, the present study elected to focus on CEPS student participants who were randomly assigned to their teachers. Additionally, this present study adopted student fixed-effects and exploited between-subject differencing, leveraging within-student variation in standardized test scores across three subjects of instruction. Analytically, the objective was to identify how student learning outcomes vary for the same student across different subjects of instruction in which teacher educational qualifications also vary, and in this analytic exercise, a key research objective was to minimize confounding bias stemming from observable and unobservable factors. 

### 2.1. China Education Panel Survey (CEPS)

Administered by the National Survey Research Center, the CEPS study is one of the first nationally representative longitudinal surveys of secondary school students in China (National Survey Research Center 2014). The CEPS study utilizes multi-stage, multi-strata, and proportional-to-size sampling (PPS) methodology in its school-based data collection design. CEPS questionnaires include a total of five surveys, designed and administered independently for students, parents, homeroom teachers, subject teachers, and principals, and contains detailed student information, such as administrative test records, in addition to student-matched family and teacher information. This present study utilized the seventh-grade cohort sample only, because the ninth-grade cohort had graduated and moved to their respective upper-secondary schools in the CEPS follow-up study. To address selection bias concerns, the analysis was further restricted to include only students who were randomly assigned to their teachers. Although previous studies have extensively evaluated the validity of class random assignment in the CEPS (see Xu and Li 2018), formal test results on randomization quality are presented in the succeeding section. 

For outcome variables, this study focused on standardized test scores in three subjects of instruction, Chinese, math, and English (range 0–150 points). A primary reason for this analytic decision was that instructional time on these subjects of instruction accounts for more than half of daily instruction, and therefore acts as key channels through which teacher quality influences student learning. Additionally, these subjects of instruction are heavily weighted in determining continuation of education beyond lower secondary, and they are considered key for foundational skills scaffolding, which bolsters tertiary education and later-life skills development opportunities. 

### 2.2. Empirical Model

Analytically, this present study built an empirical model that relates test scores on different subjects of instruction taught by different teachers of the same student to their teacher’s own level of human capital. This analytic approach allows for minimizing the influence of observable and unobservable confounding factors that do not vary across subjects of instruction, which may exist at student, teacher, or school levels. To further account for confounding influences that do vary across subjects of instruction, the empirical model includes a rich vector of subject-varying student- and teacher-level control variables. In detail, the empirical analysis is executed in the following form:

(1) $$A_{ist}=\alpha +\gamma \cdot T_{s\left(t-1\right)}+\beta \cdot X_{is\left(t-1\right)}+\pi \cdot C_{s\left(t-1\right)}+\mu_{i}+\varepsilon_{ist}$$

where $A_{ist}$ is student *i*’s follow-up test score in subject of instruction *s*. $X_{is\left(t-1\right)}$ is a vector of time-lagged student-level background characteristics for student *i* that varies across subjects of instruction at baseline. $T_{s\left(t-1\right)}$ is defined as the teacher’s level of educational attainment at baseline, who is responsible for teaching subject *s*. The model attempts to estimate an unbiased γ as the key coefficient of interest, which reflects the relationship between teachers’ own educational attainment and their contribution to student learning. 

To implement the empirical model, $\mu_{i\;}$ is the student fixed-effect and $\varepsilon_{ist}$ is the error term, and because the student fixed-effect is equivalent across subjects of instruction, its inclusion in effect controls for all subject-invariant factors. Consequently, the unit of analysis is focused on student–subject pairs, and therefore the analysis includes as many rows of observations per student as there are subjects of instruction, which results in the total analytic sample size of student–subject pairs being triple that of the number of students in the sample.

However, a key concern was that subject-varying student-level and teacher-level factors could still bias estimation of a true  γ. Therefore, the full model leverages the panel structure of the CEPS to include subject-varying student-level and teacher-level control variables, $X_{s\left(t-1\right)}$ and $C_{s\left(t-1\right)}$. On the one hand, $X_{s\left(t-1\right)}$ includes the baseline test score, participation in private tutoring, the student’s perception of the subject’s value, and the frequency of interaction with the subject teacher. On the other hand, $C_{s\left(t-1\right)}$ includes the teacher’s sex, their length of teaching experience, whether they are responsible as homeroom teacher, government certified, attended preservice training, employed on a permanent contract, hold at least a second-tier teacher rank, and received a municipal or above teaching award. In the Chinese educational setting, school principals and administrators rely on a system of teacher ranks, or *zhicheng*, and a series of teaching awards, *jiaoxuejiang*, to make hiring and promotion decisions (Chu et al. 2015). Therefore, receipts of such credentials are often used as subjective proxies for on-the-job performance, and these are relatively comparable across subjects of instruction, grade levels, and geographical regions.

### 2.3. Descriptive Statistics

Table 1 showcases a correlation matrix and descriptive statistics information for all student learning outcome variables. Firstly, the inter-subject pairwise correlation coefficients for Chinese, math, and English test scores at baseline ranged between 0.648 to 0.720 (*p*-values < 0.05), and the intra-subject pairwise correlation coefficients at baseline and follow-up ranged between 0.685 to 0.720 (*p*-values < 0.05), both indicating strong positive correlations and demonstrating good internal reliability of the test instruments. In Table 2, subject raw score information is itemized by baseline and follow-up, and results of a crude difference-in-difference estimator are computed in the final column. It is observed that across all subjects of instruction, teachers with “B.A. & Above” educational attainment were found on average to contribute 3.811 to 5.661 points more to student learning outcomes than those who do not hold such credentials, and these preliminary differences were statistically significant (*p*-values < 0.05).

In Table 3, descriptive statistics information on student-level variables (see Panel A) and teacher-level variables (see Panel B) are summarized. In Panel A, summary statistics of the study sample show 50.7 percent were female students, 67.1 percent were only children, 10.7 percent were from non-local households, 22.3 percent indicated that both parents were not living with the student, and 15.4 reported their family was facing financial hardship. For subject-varying factors, between 6.9 to 15 percent of students in the sample reported that they had attended private tutoring. Between 86.8 and 92.4 percent of students indicated that they found these subjects of instruction valuable for their careers later in life. In terms of classroom learning experiences, between 60.8 and 65.9 percent of all students reported that they felt their subject teachers were frequently interacting with them during class instruction. In Panel B, it is shown that approximately 71.2 percent of all teachers were female, close to a third (31.4 percent) of them served as homeroom teachers, most teachers (91.4 percent and 98 percent respectively) reported having attended pre-service teacher training and were government certified, whereas about two-thirds (63 percent) of all teachers reported holding permanent contracts, 87.1 percent of teachers were at Second Rank or above, and about two-fifths (43.4 percent) had received at least a municipal-level or above teaching award. The sample mean for teaching experience was 15.344 years (SD = 8.711). As for the key variable of interest, approximately 83.4 percent of teachers were found to hold a bachelor’s degree or above.

In Table 4, results of the formal test of random class assignment are shown. This test was conducted by regressing student baseline characteristics on teachers’ education attainment levels, to ascertain whether reported random class assignment was empirically valid. Importantly, regression results show that teachers’ education attainment levels were statistically unrelated with students’ baseline test scores and sex, whether they were classified as an only child, their household registration status, their participation in private tutoring, their perception of the subject’s value, and their frequency of interaction with the subject teacher (*p*-values > 0.05). While there was an indication of a negative relationship between family characteristics and teachers’ education attainment levels, it is hard to argue that these relationships had strong influences altering validity of random class assignment. Next, it was important to understand how teachers’ background characteristics varied by their levels of educational attainment. The analysis attempted to explore to what degree various observable teacher characteristics were associated with higher levels of a teacher’s own educational attainment, and the results are shown in Table 5. Results indicate that with the exception of sex, pre-service training, certification, and teacher rank, there was no statistical difference between the two groups on several dimensions of teacher characteristics. Relative to teachers without a bachelor’s degree, those with a bachelor’s degree or above were more likely to be female, trained, certified, and hold a teacher rank at or above the second-tier (*p*-values < 0.05). Finally, the analysis examined how teachers’ observable characteristics differed across subjects of instruction, and the results are shown in Table 6. Results indicate that relative to Chinese teachers, math teachers were less likely to be female and hold a teacher rank at or above the second-tier (*p*-values < 0.05), whereas English teachers were more likely to be female (*p*-values < 0.05). Altogether, the implemented tests did not detect systematic subject-specific patterns.

## 3. Results

The main analysis focused on the effect of teachers’ educational attainment levels on student learning outcomes. In Table 7, findings of the main student fixed-effect model, which directly examined the impact of teachers holding a bachelor’s degree on student learning, are presented. Sequentially, each column delineates findings from a simple model with only the key variable $T_{s\left(t-1\right)}$ in Model 1, followed by Model 2 which includes controls for subject-varying teacher-level characteristics $C_{s\left(t-1\right)}$, and Model 3 which incorporated control variables for subject-varying student-level baseline characteristics $X_{s\left(t-1\right)}$, and Model 4 was the full model with both teacher- and student-level covariates combined.

To begin, in Model 1, results of the simplest specification show that teachers’ educational attainment levels explained about 66.7 percent of the variation in between-subject student test scores, and that students tended to score higher when teachers with a bachelor’s degrees and above taught their respective subjects; the associated impact was 0.061 standard deviations (*p*-value < 0.05). Next, in Models 2 and 3, results indicate a statistically significant impact of 0.056 standard deviations (*p*-value < 0.05) after accounting for teacher-level covariates, and 0.058 standard deviations (*p*-value < 0.05) after adding student-level covariates. In the full specification with both student- and teacher-level covariates simultaneously considered, there is a statistically significant effect of 0.069 standard deviations (*p*-value < 0.05), as indicated in Model 4. In other words, holding all else equal, having a teacher that was at least bachelor-educated was found to increase students’ learning outcomes by as much as 0.069 standard deviations, with a 95 percent confidence interval ranging between 0.028 to 0.110 standard deviations.

As a final step to examine heterogeneity of effects, teachers’ educational attainment levels, $T_{s\left(t-1\right)}$, were interacted with student baseline characteristics to assess whether the contribution of teacher educational attainment on student learning varied for different categories of students. These grouping variables included the student’s sex, whether they were the only child, whether their baseline test score was one standard deviation (SD) below the sample mean, their holding of a non-local household registration, whether they identified as a “left-behind child” who was not living with either of their parents, and whether they self-reported being in financial hardship. In reference to Equation (1), the inclusion of an interaction term changes the interpretation of γ, which now refers to the impact of a teacher’s educational attainment on learning outcomes among the “advantaged” student group, while the coefficient for the interaction term corresponds to the degree in which learning outcomes of “disadvantaged” students are differentially affected by teachers’ educational attainment. In Table 8, detailed results show that all interaction term coefficients remained statistically insignificant at the 0.05 level, suggesting that there was no discernible difference between the disadvantaged and advantaged student groups in terms of effects, implying that better educated teachers can improve student learning for a broad base of students.

## 4. Discussion and Conclusions

This present study set out to answer the research question of whether better educated teachers contribute more to students’ cognitive development than those with lower educational attainment, and examined whether such effects apply equitably across students of different backgrounds. By employing student fixed-effect modelling, the empirical analysis related differences in teachers’ educational attainment levels across three subjects of instruction to the respective variation in student test scores, and findings confirmed that students benefited cognitively from having better educated teachers.

This positive effect was evaluated as corresponding to 0.069 standard deviations per academic year, and was consistent for students from different backgrounds. To interpret the size of this effect in relation to broader educational context, it is useful to reference findings from previous studies: for instance, OECD (2016) reported 0.25 to 0.30 standard deviations as the amount of expected learning gain per year among middle-to-high income countries, while Evans and Yuan (2019) estimated learning gains per year to be between 0.15 to 0.21 standard deviations among low-to-middle income countries. More recently, new evidence by Avvisati and Givord (2021) found that average expected learning gains in the Beijing, Shanghai, Jiangsu, and Zhejiang regions of China fell between 0.16 to 0.21 standard deviations, depending on the subject of instruction.

Based on these prior estimates, conservative calculations indicate that the estimated effect sizes of 0.069 standard deviations increase in student learning outcomes approximately corresponds to two additional months of extra learning in a typical nine-month school year. In other words, findings in this present study suggest that a better educated teacher, who holds a bachelor’s degree or above, can be about 20 percent more productive at improving student learning than a less educated teacher. While this present study identified a small-to-medium effect size (0.069 standard deviations) in student learning gains being attributable to better educated teachers, the longer-term aggregate learning gains could be substantial, considering that such effects are likely to compound over multiple years, and are likely to benefit an entire class of students if they are consistently exposed to more qualified teachers.

In a broader scope, findings in this present study coincide with new evidence that demographic transition in many countries is spurring increased demand for teachers (Crawfurd and Pugatch 2020), and illustrate the immense value of improving teacher education and bolstering a teacher’s own level of human capital development in order to achieve quality and equitable education. For one, the cognitive returns from investing in teachers, either through training, recruiting, or retaining more qualified individuals to teach, can be considerable for students. Evidence confirms that better educated teachers are more effective in leading and teaching in the classroom. For another, findings show that students from a wide spectrum of socioeconomic backgrounds benefit equally from better educated teachers, which makes such policies both education quality-enhancing and equitably beneficial. In this regard, policies targeting teacher education improvements may represent a useful welfare enhancing reform tool to promote social justice by building more quality and inclusive educational institutions, particularly when broader societal uncertainties are reshaping the future of how students access learning opportunities (Liu 2021b). Additionally, school personnel management practices may consider a stronger emphasis on retaining qualified and effective teachers, because when they do leave, the void of unrealized student cognitive development can be considerable (Liu 2021a).

On the whole: findings in the present study contribute new evidence to a large cluster of studies highlighting the importance of teacher education (Liu and Xie 2021) and that more qualified individuals are also more likely to be competent and effective in meeting a variety of instructional needs (Loeb et al. 2014). While some scholars question the usefulness of policies aimed at improving teacher education and increasing teacher educational attainment, findings in this present study refute such doubts by illuminating the significant and substantive cognitive returns associated with having better educated teachers. Nonetheless, understanding how a teacher’s own human capital development translates practically into more effective teaching is still a research gap, and future studies are needed to better unpack the complexity of teaching and to understand intricate links between teacher education and classroom instruction. Finally, it is also essential to determine whether the positive influence of teacher education holds for subjects beyond those analyzed in the present study, such as in praxis-oriented subjects of instruction, including arts, music, and physical education, which would further the knowledge base on how to effectively support teacher education and instructional preparation.

## Figures and Tables

**Table 1:** Correlation matrix of baseline and follow-up test scores.

Test Score Variables		1	2	3	4	5	6
1	Baseline Chinese	1					
2	Baseline Math	0.648 *	1				
3	Baseline English	0.720 *	0.705 *	1			
4	Follow-up Chinese	0.720 *	0.539 *	0.613 *	1		
5	Follow-up Math	0.579 *	0.685 *	0.607 *	0.679 *	1	
6	Follow-up English	0.570 *	0.596 *	0.712 *	0.662 *	0.730 *	1
N		3436	3436	3436	3436	3436	3436
Mean		76.531	73.933	81.617	79.626	72.243	68.202
SD		18.661	29.162	26.703	22.608	33.139	30.449
Max		126	150	150	142	150	149
Min		1	3	4	0	0	0

Note: Rows 1–6 present Pearson’s R correlation coefficients. * denotes *p*-value < 0.05.

**Table 2:** Baseline and follow-up test scores by teacher educational attainment (N = 3436).

	B.A. & Above		Below B.A.		Difference-in-Difference Estimator {(2) − (1)} − {(4) − (3)}
	Baseline (1)	Follow-Up (2)	Baseline (3)	Follow-Up (4)	
Chinese	76.072	80.651	80.773	80.421	4.931 *
Math	73.752	72.665	77.798	72.900	3.811 *
English	81.372	69.719	83.890	66.576	5.661 *

Note: * denotes *p*-value < 0.05.

**Table 3:** Descriptive statistics of study participants (N = 3436).

	Definition and Metrics	N	Mean	Max	Min
Panel A: Student Variables					
Female	Female = 1, male = 0	3436	0.507	1	0
Only Child	Only child = 1, otherwise = 0	3436	0.671	1	0
Non-local	Non-local household registration = 1, otherwise = 0	3436	0.107	1	0
Both parents not living with student	Both parents not living with student = 1, at least one parent living with student = 0	3436	0.223	1	0
Family facing financial hardship	Family facing financial hardship = 1, otherwise = 0	3436	0.154	1	0
Private Tutoring	Enrolled in private tutoring = 1, otherwise = 0				
Chinese		3436	0.069	1	0
Math		3436	0.127	1	0
English		3436	0.150	1	0
Subject Valuable	Found subject valuable = 1, otherwise = 0				
Chinese		3436	0.924	1	0
Math		3436	0.900	1	0
English		3436	0.868	1	0
Teacher Interaction	Interacted frequently with teacher during instruction = 1, otherwise = 0				
Chinese		3436	0.634	1	0
Math		3436	0.608	1	0
English		3436	0.659	1	0
Panel B: Teacher Variables					
Female	Female = 1, Male = 0	253	0.712	1	0
Homeroom	Homeroom teacher = 1, otherwise = 0	253	0.314	1	0
Pre-Service Training	Attended pre-service training = 1, otherwise = 0	253	0.914	1	0
Certification	Government certified = 1, otherwise = 0	253	0.980	1	0
Contract	Employed on permanent contract = 1, otherwise = 0	253	0.630	1	0
Teacher Rank	Hold second-tier or above teacher rank = 1, otherwise = 0	253	0.871	1	0
Teaching Award	Received municipal or above teaching award = 1, otherwise = 0	253	0.434	1	0
Teaching Experience	Experience in years	253	15.344 (8.711)	39	0
B.A. & Above	Bachelor’s & above = 1, otherwise = 0	253	0.834	1	0

Note: Standard deviation in parenthesis, where appropriate.

**Table 4:** Test of random class assignment (N = 10,308).

Dependent Variables	B.A. & Above (Reference = Below B.A.)
Baseline Test Score (SDs)	0.059
	(0.047)
Female	0.034
	(0.073)
Only Child	0.433
	(0.306)
Non-local	−0.038
	(0.105)
Both parents not living with student	−0.286 *
	(0.128)
Family facing financial hardship	−0.255 *
	(0.101)
Private Tutoring	0.237
	(0.292)
Subject Valuable	0.027
	(0.105)
Teacher Interaction	−0.031
	(0.101)

Note: Each row represents an independent regression. All coefficients are from probit regression, with exception of baseline test score, which is from OLS regression. Robust standard errors clustered at the school level and in parenthesis, * denotes *p*-value < 0.05.

**Table 5:** Teacher characteristics by educational attainment (N = 253).

Dependent Variables	B.A. & Above (Reference = Below B.A.)
Female	0.835 *
	(0.232)
Homeroom	0.192
	(0.158)
Pre-Service Training	0.632 *
	(0.243)
Contract	0.122
	(0.198)
Certification	0.951 *
	(0.303)
Teacher Rank	0.747 *
	(0.235)
Teaching Award	0.144
	(0.230)
Teaching Experience (years)	−2.805
	(1.818)

Note: Each row represents an independent regression. All coefficients are from probit regression, with exception of teaching experience, which is from OLS regression. Robust standard errors clustered at the school level and in parenthesis, * denotes *p*-value < 0.05.

**Table 6:** Teacher characteristics by subject of instruction (N = 253).

Dependent Variables	Subject of Instruction (Reference = Chinese)	
	Math	English
B.A. & Above	−0.017	−0.008
	(0.196)	(0.242)
Female	−0.462 *	0.592 *
	(0.172)	(0.225)
Homeroom	0.328	0.361
	(0.231)	(0.263)
Pre-Service Training	−0.123	−0.626
	(0.307)	(0.377)
Contract	−0.340	−0.225
	(0.199)	(0.241)
Certification	0.140	0.588
	(0.410)	(0.489)
Teacher Rank	0.445 *	−0.077
	(0.177)	(0.153)
Teaching Award	0.107	−0.151
	(0.182)	(0.183)
Teaching Experience (years)	1.921	0.143
	(1.113)	(1.188)

Note: Each row represents an independent regression. All coefficients are from probit regression, with exception of teaching experience, which is from OLS regression. Robust standard errors clustered at the school level and in parenthesis, * denotes *p*-value < 0.05.

**Table 7:** Effect of teacher educational attainment on student learning outcomes.

Dependent Variable: Follow-Up Test Scores (SDs)	Model (1)	Model (2)	Model (3)	Model (4)
B.A. & above (reference = Below B.A.)	0.061 *	0.056 *	0.058 *	0.069 *
	(0.019)	(0.020)	(0.018)	(0.021)
Adjusted R-squared	0.667	0.667	0.720	0.718
Student-Level Covariates	No	No	Yes	Yes
Teacher-Level Covariates	No	Yes	No	Yes
Student Fixed-Effects	Yes	Yes	Yes	Yes
Observations	10,308	10,308	10,308	10,308

Note: Robust standard errors presented in parenthesis, * denotes *p*-value < 0.05.

**Table 8:** Heterogeneous impact of educational attainment on student learning outcomes.

Dependent Variable: Follow-Up Test Scores (SDs)		Model (5)	Model (6)	Model (7)	Model (8)	Model (9)	Model (10)
Teacher-level Variables							
X	BA or above (below = 0)	0.051 *	0.061 *	0.056 *	0.050 *	0.064 *	0.047 *
		(0.024)	(0.021)	(0.019)	(0.011)	(0.024)	(0.021)
Interaction Terms							
	X * Female (male = 0)	0.004					
		(0.036)					
	X * Only Child (otherwise = 0)		−0.046				
			(0.042)				
	X * Low baseline score (otherwise = 0)			−0.038			
				(0.026)			
	X * Non-local (otherwise = 0)				−0.029		
					(0.057)		
	X * Both parents not living with student (otherwise = 0)					−0.017	
						(0.046)	
	X * Family facing financial hardship (otherwise = 0)						0.016
							(0.044)
Adjusted R-squared		0.718	0.721	0.721	0.719	0.718	0.720
Student-Level Covariates		Yes	Yes	Yes	Yes	Yes	Yes
Teacher-Level Covariates		Yes	Yes	Yes	Yes	Yes	Yes
Student Fixed-Effects		Yes	Yes	Yes	Yes	Yes	Yes
Observations		10,308	10,308	10,308	10,308	10,308	10,308

Note: Robust standard errors in parenthesis, * denotes *p*-value < 0.05.

## Data Availability

China Educational Panel Survey (CEPS) dataset is publicly-available at https://doi.org/10.18170/DVN/KURJUU.
